# Supplementary material for: Stacking Model‐Based Classifiers for Dealing With Multiple Sets of Noisy Labels
Source: Biom J. 2025 Mar 12;67(2):e70042. doi: 10.1002/bimj.70042 (PMC11898607; doi:10.1002/bimj.70042)
Supplement: Supplementary file 1 — Supporting Information [file BIMJ-67-e70042-s001.zip › density-based-ensemble-model-main/competitors_real_data/netlab-master/netlab-master/help/demprior.htm]

Netlab Reference Manual demprior


# demprior

## Purpose

Demonstrate sampling from a multi-parameter Gaussian prior.

## Synopsis

```
demprior
```

## Description

This function plots the functions represented by a multi-layer perceptron
network when the weights are set to values drawn from a Gaussian prior
distribution. The parameters `aw1`, `ab1` `aw2` and `ab2`
control the inverse variances of the first-layer weights, the hidden unit
biases, the second-layer weights and the output unit biases respectively.
Their values can be adjusted on a logarithmic scale using the sliders, or
by typing values into the text boxes and pressing the return key.

## See Also

`mlp`

---

**Pages:**
Index


---

Copyright (c) Ian T Nabney (1996-9)
